# Chaotic universe model

**DOI:** 10.1038/s41598-017-18681-4

**Published:** 2018-01-15

**Authors:** Ekrem Aydiner

**Affiliations:** Department of Physics, Faculty of Science, Istanbul University, Istanbul, 34134 Turkey

## Abstract

In this study, we consider nonlinear interactions between components such as dark energy, dark matter, matter and radiation in the framework of the Friedman-Robertson-Walker space-time and propose a simple interaction model based on the time evolution of the densities of these components. By using this model we show that these interactions can be given by Lotka-Volterra type equations. We numerically solve these coupling equations and show that interaction dynamics between dark energy-dark matter-matter or dark energy-dark matter-matter-radiation has a strange attractor for 0 > w_*de*_ >−1, *w*_*dm*_ ≥ 0, *w*_*m*_ ≥ 0 and *w*_*r*_ ≥ 0 values. These strange attractors with the positive Lyapunov exponent clearly show that chaotic dynamics appears in the time evolution of the densities. These results provide that the time evolution of the universe is chaotic. The present model may have potential to solve some of the cosmological problems such as the singularity, cosmic coincidence, big crunch, big rip, horizon, oscillation, the emergence of the galaxies, matter distribution and large-scale organization of the universe. The model also connects between dynamics of the competing species in biological systems and dynamics of the time evolution of the universe and offers a new perspective and a new different scenario for the universe evolution.

## Introduction

The formation, structure, dynamics and evolution of the universe has always been of interest. It is commonly accepted that modern cosmology began with the publication of Einstein’s seminal article in 1917^1^. Applying the general relativity to the entire universe, Einstein suggested that the universe was static, and spatially curved. Following from this, to explain the structure and dynamics of the universe many interesting models based on Einstein model have been proposed such as flat and expanding universe^2^, expanding flat space model, spherical and hyperbolic expanding space^3^, original big-bang model^4–6^, expanding flat space^7^, kinematic expanding models^8^, oscillating or cyclic universe models^9,10^, buble universe and inflation bubble universe models^9–12^, chaotic inflation model^13^ etc. Amongst these models, the big-bang model has been the most accepted one. This is due to the cosmic microwave background (CMB), and cosmic red shift discovered by Hubble observations as well as observations confirming the abundance of light elements in the universe supporting the big-bang scenario. However, new experimental findings such as Type Ia supernovae (SNIa) data^14–17^, CMB anisotropy^18,19^, and large scale structure (LSS)^20–22^, showing that the universe does not only expand but does this with an acceleration makes this cosmic scenario more exciting. There is no explanation to this expansion with an acceleration yet. Cosmologists are still working on new models and scenarios to address this situation. One of the best scenarios attempting this is the dark energy. Unfortunately, there is no confirmation of the physical source of this dark energy. Although the origin of the dark energy is not known yet, it is well known that mater is not the only ingredient of the universe. According to what is known today, at present, the universe is composed of approximately 75% dark energy, 20% cold dark matter, 5% baryonic matter and negligible amount of radiation^14–22^. To explain the nature of the dark energy, there are various dark energy models and mechanisms such as the cosmological constant Λ (vacuum or dark energy) proposed. The cosmological constant-cold dark matter-matter model (ΛCDM) works very well and is in agreement with a large number of recent observations. However, to state without considering highly hypothetical models and their problematic propositions, some of the questions to be answered are the singularity, cosmic coincidence, big crunch, big rip, horizon, oscillation, emergence of the galaxies, matter distribution and large scale organization of the universe. Despite the great success of the modern cosmology, it is obvious that there can be no success in the development of an integrated theory on the dynamics and evolution of the universe without answering these questions.

There are many proposed models attempting to answer questions above mentioned emerge from the big-bang and other theories based on the theory of the modern cosmology founded by Einstein. For example, oscillating or cyclic universe models, and dark-matter interaction model were proposed to solve the singularity, and fine tuning problems respectively gaining importance in the field. However, no theoretical relationship has been established between the cyclic universe and the dark energy-dark matter interaction models so far, sufficiently addressing the evolution and dynamics of the universe. Questions on the past and future evolution of the universe, and the mechanisms of the dynamics stemming from this evolution have not sufficiently been addressed. But until now, a more comprehensive scenario has not been developed that addresses the evolution of the universe and the existing cosmological problems. Work based on the interaction of dark energy and dark matter seems to bring optimism to the field^23–48^. This is because the presence of such interactions may have hints that may help to understand the dynamics of the universe leading to a the development of a more realistic scenario. In this work, in contrast to well known popular models, it is assumed that there is a non-linear interaction between dark energy, dark matter, matter and radiation. Although somehow hypothetical, this assumption may have the potential of solving many important problems of cosmology such as the singularity, cosmic coincidence, big crunch, big rip, horizon, oscillation, emergence of galaxies, matter distribution and large scale organization of the universe.

The idea of presence of a non-linear interaction between dark energy, dark matter, matter and radiation may enable the development of a new cosmology scenario on the evolution and dynamics of the universe. In this work, the interactions between components forming the universe is modeled and possible outcomes are discussed. This model is a novel one. The interaction models are based on Friedman-Robertson-Walker (FRW) framework leading to investigation of possible dynamics. We believe in the importance of this work because of the following points: Firstly, the non-linear interaction between the components of the universe were first given by the Lotka-Volterra type equations^49,50^. It is known that the Lotka-Volterra equation and its variations are mathematical models proposed to model the competition between biological species. It is interesting that the Lotka-Volterra type equation written for cosmology is in the simplest differential form. Secondly, the Lotka-Volterra type equations written for cosmology can give chaotic solutions depending on the values of the parameters of interaction. This is an important outcome for cosmology carrying a potential in helping us to understand questions mentioned above using the non-linear interaction dynamic. Furthermore, the model proposed here combines the big-bang and oscillating universe models in a different way and a perspective.

## Results

### Interaction between dark energy - dark matter

Firstly we present the theoretical results in which we show that the interaction between dark energy and dark matter can be given a coupling equation likes Lotka-Volterra. In order to obtain the coupling interaction equation we follow belove theoretical procedure. Based on the Friedman-Robertson-Walker metric, the interaction between dark matter and dark energy can be given as follows:

1a $${\dot{\rho }}_{de}+3H({\rho }_{de}+{p}_{de})=-Q$$

1b $${\dot{\rho }}_{dm}+3H({\rho }_{dm}+{p}_{dm})=Q$$

where *Q* is arbitrary coupling function and subscript stands for a generic dark energy model to be specified^23–41^. The conservation equations are subject to the Friedman constraint

2 $${H}^{2}=\frac{{\kappa }^{2}}{3}({\rho }_{de}+{\rho }_{dm}),\quad \dot{\rho }H\,=-\frac{{\kappa }^{2}}{2}({\rho }_{de}+{p}_{de}+{\rho }_{dm}+{p}_{dm}).$$

In this situation, the total energy conservation holds,

3 $${\dot{\rho }}_{eff}+3H({\rho }_{eff}+{p}_{eff})=0$$

where *ρ*_*eff*_ = *ρ*_*de*_ + *ρ*_*dm*_ and *p*_*eff*_ = *p*_*de*_ + *p*_*dm*_ and the Friedman-Robertson-Walker Eq. (2) do change. In this model it is assumed that dark matter has a pressure. Assuming that dark matter has no pressure is purely hypothetical. Furthermore, the assumption of zero pressure contradicts the idea of the change in density. Recently, it is suggested that dark matter has a pressure and various mechanisms have been proposed for this pressure^51^. In this work, it can be suggested that the dark matter’s contribution to pressure may be caused by volume exclusion just like the van der Walls gas. That’s why the pressure of dark matter should not be zero. However small, the nonzero value of dark matter means that its EoS parameters should be nonzero too. In other words, for *p*_*dm*_ > 0 the EoS parameter for dark matter can be given as *w*_*dm*_ > 0. On the other hand, it is known that the form of *Q* is determined under phenomenological assumptions, mainly, the dimensional analysis is used to construct interactions. It is reasonable to consider interactions which could improve previously known results and at the same time will not make the mathematical treatment of the problems complicated. It is widely believed that deeper understanding of the nature of dark energy and dark matter could give fundamental explanations of the phenomenological assumptions about interaction. There are different *Q* definition in the literature^23–45^. A simple interaction coupling can be chosen as *Q* = ±*γρ*_*dm*_*ρ*_*de*_. In this case, these questions can then be expressed as

4a $${\dot{\rho }}_{de}+3H({\rho }_{de}+{p}_{de})=-\gamma {\rho }_{dm}{\rho }_{de}$$

4b $${\dot{\rho }}_{dm}+3H({\rho }_{dm}+{p}_{dm})=\gamma {\rho }_{dm}{\rho }_{de}$$

if *γ* > 0, the interaction suggests that dark matter is converted into dark energy, while *γ* < 0 suggests the inverse process^52^. By setting *p*_*de*_ = *ρ*_*de*_*w*_*de*_ and *p*_*dm*_ = *ρ*_*dm*_*w*_*dm*_ these equations are given by

5a $$\frac{d{\rho }_{de}}{dt}=-3H(1+{w}_{de}){\rho }_{de}-\gamma {\rho }_{de}{\rho }_{dm}$$

5b $$\frac{d{\rho }_{dm}}{dt}=\gamma {\rho }_{dm}{\rho }_{de}-3H(1+{w}_{dm}){\rho }_{dm}.$$

Now we can write *r*_1_ = −3*H*(1 + *w*_*de*_) > 0 for *w*_*de*_ < −1, and *r*_2_ = 3*H*(1 + *w*_*dm*_) > 0 for *w*_*dm*_ ≥ 0. Hence we obtain

6a $$\frac{d{\rho }_{de}}{dt}={r}_{1}{\rho }_{de}-\gamma {\rho }_{de}{\rho }_{dm}$$

6b $$\frac{d{\rho }_{dm}}{dt}=\gamma {\rho }_{dm}{\rho }_{de}-{r}_{2}{\rho }_{dm}.$$

By using these relation

7 $${x}_{1}=\frac{\gamma }{{r}_{2}}{\rho }_{de}\,,\quad {x}_{2}=\frac{\gamma }{{r}_{1}}{\rho }_{dm}$$

for a constant or very slowly changing Hubble parameter *H*, Eq. (5) with help of Eqs (6) and (7) can be transformed to

8a $$\frac{d{x}_{1}}{dt}={r}_{1}{x}_{1}(1-{x}_{2})$$

8b $$\frac{d{x}_{2}}{dt}={r}_{2}{x}_{2}({x}_{1}-1)$$

where *r*_1_ > 0 for *w*_*de*_ < −1 and *r*_2_ > 0 for *w*_*dm*_ ≥ 0. As it can be seen in Eq. (8), the choice of the interaction term leads us to Lotka-Volterra type equations. This equation is a our main result. To recall, Lotka-Volterra equations^49,50^ represent the competition between two species and they are used widely in biology, chemistry and various other fields. In this work, the interaction equations between dark energy and dark matter of cosmological systems are used corresponding to the competing prey and predator species in biology.

The dynamic and stability analysis of the Eq. (8) are given belove. The model reaches equilibrium when both of the derivatives are equal to zero.

9a $${r}_{1}(1-{x}_{2}){x}_{1}=0$$

9b $${r}_{2}({x}_{1}-1){x}_{2}=0.$$

When solved for *x*_1_ and *x*_2_ the above system of equations yields

10a $${S}_{1}\,:=\{{x}_{1},{x}_{2}\}=\{0,0\}$$

10b $${S}_{2}\,:=\{{x}_{1},{x}_{2}\}=\{1,1\}.$$

These are fixed points of the coupling equations. The stability of the fixed points at the origin can be determined by performing a linearisation by using partial derivation. The Jacobian matrix of the model is

11 $$J=(\begin{array}{cc}{r}_{1}(1-{x}_{2}) & -{r}_{1}{x}_{1}\\ {r}_{2}{x}_{2} & {r}_{2}({x}_{1}-1)\end{array})$$

where *J* = *J* (*x*_1_, *x*_2_). When evaluated at the steady state of (0, 0) the Jacobian matrix *J* becomes

12 $$J(\mathrm{0,0})=(\begin{array}{cc}{r}_{1} & 0\\ 0 & -{r}_{2}\end{array})$$

For fixed point *S*_1_ = (0, 0), the eigenvalues of this matrix are *λ*_1_ = *r*_1_ = −3*H* (1 + *w*_*de*_) for *w*_*de*_ <−1 and *λ*_2_ = −*r*_2_ = −3*H*(1 + *w*_*dm*_) for *w*_*dm*_ ≥ 0. Evaluating *J* at the second fixed point leads to

13 $$J(1,1)=(\begin{array}{cc}0 & -{r}_{1}\\ {r}_{2} & 0\end{array}).$$

For fixed point *S*_2_ = (1, 1), the eigenvalues are ${\lambda }_{1}=3iH\sqrt{{w}_{dm}+1}\sqrt{{w}_{de}+1}$ and ${\lambda }_{2}=-3iH\sqrt{{w}_{dm}+1}\sqrt{{w}_{de}+1}$ in the case of *w*_*de*_ < −1 and *w*_*de*_ ≥ 0. In this model the value of eigenvalues depend on EoS parameters *w*_*dm*_ and *w*_*de*_. Therefore characteristic properties of these eigenvalues are determined by sign of EoS parameters. The stability of these fixed point are of significance. For the fixed point *S*_1_ has two real eigenvalues. The relation between eigenvalues is given as *λ*_2_ > 0 > *λ*_1_ which indicates fixed point *S*_1_ is a saddle point. However, the fixed point *S*_2_ has two nonzero imaginary eigenvalues as ${\lambda }_{1,2}=\pm 3iH\sqrt{{w}_{dm}+1}\sqrt{{w}_{de}+1}$ for *w*_*de*_ < −1. Hence the linear analysis cannot tell more about nature of *S*_2_, since the eigenvalues may have null real part. Therefore to understand better how the *limit cycles* behave and on what they do depend on, let’s take the ratio of the two equation of the models, hence trying to solve

14 $$\frac{d{x}_{2}}{d{x}_{1}}=\frac{{r}_{2}{x}_{2}({x}_{1}-1)}{{r}_{1}{x}_{1}(1-{x}_{2})}$$

by separating the variables, the equation reads:

15 $$\frac{{r}_{1}(1-{x}_{2})}{{x}_{2}}d{x}_{2}=\frac{{r}_{2}({x}_{1}-1)}{{x}_{1}}d{x}_{1}$$

integrating now from an arbitrary initial point (*x*_10_, *x*_20_) and an arbitrary point (*x*, *y*), one obtains

16 $${r}_{1}(\mathrm{ln}\,{x}_{2}-{x}_{2})+{r}_{2}(\mathrm{ln}\,{x}_{1}-{x}_{1})=F({x}_{1},{x}_{2})$$

where *F*(*x*_1_, *x*_2_) is the equation of a surface, which depends on the initial conditions (*x*_10_, *x*_20_) and represents an invariant of motion. Analyzing Hessian matrix *H* (*F*), one can show that *F*(*x*_1_, *x*_2_) is a convex function of (*x*_1_, *x*_2_), that *S*_2_ represents the critical points, and that the contour lines are close curves. These close curves ones are also the *limit cycles* of the system Eq. (8), and all the trajectories go onto them.

**Figure 1:**
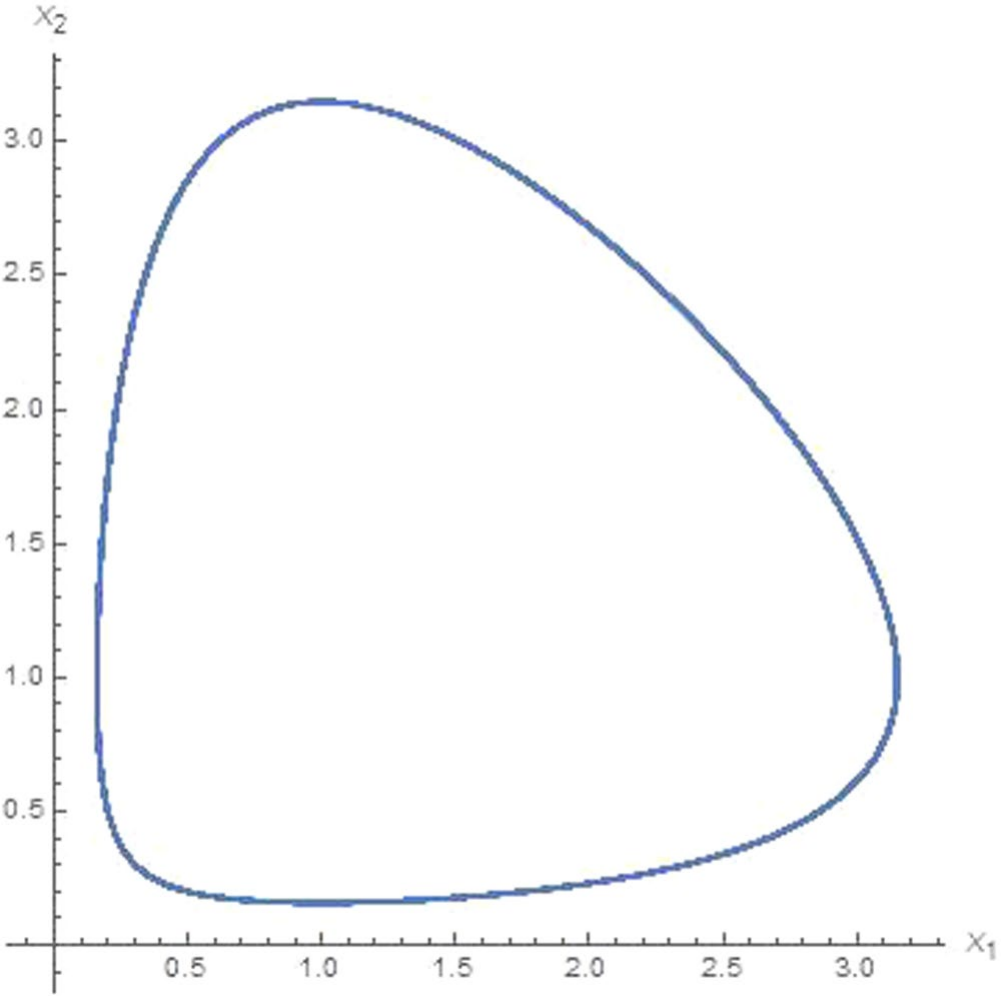
Dimensionless dark matter density *x*_1_ and dark energy density *x*_2_ for 1000 time step. The parameters are set as *r*_1_ = 1.0, *r*_2_ = 1.0.

It must be noted that for *w*_*dm*_ < −1 and *w*_*de*_ < −1 the eigenvalues for *S*_2_ = (1, 1) are be real, hence *S*_2_ is the saddle point. For this condition, the trajectory of dynamic will not be close. This means that, since there will be no cyclic relationship between the two, dark matter and dark energy of the universe will be rapidly reduced to zero or one will be transformed to the other one and disappear completely. Theoretical and observational data indicates that approximately 5% of the universe is formed of matter and hence any dynamic that causes the disappearance of dark matter cannot be correct. In fact this model establishes lower and upper boundaries for the EoS parameters of dark energy. Assuming that the interaction equations are correct, for a positive and finite value for the dark matter pressure the EoS parameter is given as *w*_*dm*_ > 0, and for negative pressure value of dark energy and cyclic relationships the EoS value should be *w*_*de*_ < −1. Another point to be noted is the following: Here, the interaction parameter is chosen as *Q* = *γρ*_*dm*_*ρ*_*de*_. This implies that the transformation between dark energy and dark matter will be equal for both directions. However, a nonlinear transformation approach will be more realistic. The interaction parameters may be chosen different types. This has also been considered in our research but not presented here. The findings show that a different interaction parameter does not affect the characteristic of the solution. This is because of the fact that the interaction parameters lie in the eigenvalues determining the characteristics of the fixed point. A simple numerical solution of Eq. (8) is given in a phase space. Figure 1 shows the interchanges of the densities of dark energy *x*_1_ and dark matter *x*_2_ for *r*_1_ = 1.0, *r*_2_ = 1.0. This cycle trajectory is independent of the interaction parameters between dark matter and dark energy but depends on EoS parameters. Cycle solutions are obtained for all *r*_1_ > 0 and *r*_2_ > 0 values. These conditions can be satisfied when taking *w*_*de*_ < −1 for dark energy and *w*_*dm*_ ≥ 0 for dark matter.

Here we examine the dynamics of dark energy density versus dark matter density. As it can be seen from Fig. 1, Eq. (8) has a closed cyclic trajectory solution for arbitrary parameters *r*_1_ = 1.0, *r*_2_ = 1.0. We mentioned above that the characteristic of the trajectory completely depends on eigenvalues ${\lambda }_{1}=3iH\sqrt{{w}_{dm}+1}\sqrt{{w}_{de}+1}$ and ${\lambda }_{2}=-3iH\sqrt{{w}_{dm}+1}\sqrt{{w}_{de}+1}$ instead of the parameters *r*_1_ and *r*_2_ since they directly determine the nature of the fixed points. On the other hand, the Hubble parameter *H* behaves as a scaling parameter and it equally affects the time evolution of the densities. As it can be seen from Eq. (14) that the Hubble parameters in *r*_1_ and *r*_2_ are canceled at the both side of the Eq. (14). Therefore, we have to note that the interactions are independent of *H*.

### Quadratic interactions in dark sector

Here we present contribution of the quadratic interaction terms to the dynamics of the dark energy and dark matter. In the previous subsection by using Eq. (32) we set EoS parameters as *p*_*dm*_ = *ρ*_*dm*_*w*_*dm*_, *p*_*de*_ = *ρ*_*de*_*w*_*de*_ for the dark matter and dark energy. In Eq. (32) *p* = *ρw* is valid ideal fluid when it is homogeneously distributed in a volume. This means that pressure at every point of the *V*. Whereas, in realistic systems, pressure may not homogeneously distribute in the volume. In this case pressure *p* can be written as *p* = *p*(*ρ*) in terms of *ρ*

17 $$p=p(\rho )=\sum _{n=0}^{N}{A}_{n}{\rho }^{n}={p}_{0}+{A}_{1}\rho +{A}_{2}{\rho }^{2}+\ldots \,.$$

This form of pressure is known as a perturbative pressure or barotropic EoS (See ref.^53^). Here we interested in the quadratic form of the EoS *p* = *p*_0_ + *A*_1_*ρ* + *A*_2_*ρ*^2^ by ignoring higher order terms. The usual scenario for a cosmological fluid is a standard linear EoS (*p*_0_ = *A*_2_ = 0), in which case *A*_1_ = *w* is usually restricted to the range between ±1. In a high energy, regime restricted equation of state can be chosen as *p* = *A*_1_*ρ* + *A*_2_*ρ*^2^ where the parameter *A*_2_ set the characteristic energy scale of the quadratic term^53^.

In order to obtain more information about nature of the coupling interactions, equation of states can be modified as ${p}_{de}={A}_{1de}{\rho }_{de}+{A}_{2de}{\rho }_{de}^{2}$ and ${p}_{dm}={A}_{1dm}{\rho }_{dm}+{A}_{2dm}{\rho }_{dm}^{2}$ for inhomogeneous distributed dark energy and dark matter. By setting *A*_1*de*_ = *w*_*de*_, ${A}_{2de}={w^{\prime} }_{de}$ and *A*_1*dm*_ = *w*_*dm*_, ${A}_{2dm}={w^{\prime} }_{dm}$, the Eq. (5a) and (5b) can be reorganized as

18a $$\frac{d{\rho }_{de}}{dt}=-3H(1+{w}_{de}){\rho }_{de}-3H{w^{\prime} }_{de}{\rho }_{de}^{2}-\gamma {\rho }_{de}{\rho }_{dm}$$

18b $$\frac{d{\rho }_{dm}}{dt}=\gamma {\rho }_{de}{\rho }_{dm}-3H(1+{w}_{dm}){\rho }_{dm}-3H{w^{\prime} }_{dm}{\rho }_{dm}^{2}$$

By using Eq. (7), these equation can be transformed to

19a $$\frac{d{x}_{1}}{dt}={r}_{1}{x}_{1}(1-{x}_{2})-{r^{\prime} }_{1}{x}_{1}^{2}$$

19b $$\frac{d{x}_{2}}{dt}={r}_{2}{x}_{2}({x}_{1}-1)-{r^{\prime} }_{2}{x}_{2}^{2}$$

where *r*_1_ = −3*H* (1 + *w*_*de*_) > 0 for *w*_*de*_ < −1 and *r*_2_ = 3*H* (1 + *w*_*dm*_) > 0 for *w*_*dm*_ ≥ 0 as well in the previous section. Additionally $${r^{\prime} }_{1}=-9{H}^{2}{w^{\prime} }_{de}(1+{w}_{dm}){\gamma }^{-1} > 0$$ for $${w^{\prime} }_{de} < -1$$ and *w*_*dm*_ > 0 values, and $${r^{\prime} }_{2}=-9{H}^{2}{w^{\prime} }_{dm}$$$$(1+{w}_{de}){\gamma }^{-1} > 0$$ for *w*_*dm*_ > 0 and *w*_*de*_ < −1 values. As can be seen that the quadratic term in rhs of Eq. (19a) and (19b) correspond to self-interacting terms in between components of the universe^54–59^. One can clearly see that these equations are coupled two interacting species such as dark matter and dark energy in the universe and which looks like Lotka-Volterra equations. As a result, by using quadratic EoS we find that coupling interactions lead to self-interacting Lotka-Volterra equations. Our purpose here is to show the contribution of the quadratic terms to the dark energy and dark matter interaction. Here we have only studied the dark energy and the dark matter, however, this discussion can be extended to the number of the higher components.

Now we can briefly give the stability analysis of the fixed points. The two equation of the self-interacting models in the phase plane is given

20 $$\frac{d{x}_{2}}{d{x}_{1}}=\frac{{x}_{2}({r}_{2}+{r^{\prime} }_{2}{x}_{2}+{r}_{2}{x}_{1})}{{x}_{1}({r}_{1}+{r^{\prime} }_{1}{x}_{1}-{r}_{1}{x}_{2})}.$$

Unlike Eq. (14), the phase plane differential equation in Eq. (20) is not separable. The simple isoclines are

21a $$\frac{d{x}_{2}}{d{x}_{1}}=0\to {r^{\prime} }_{1}{x}_{1}-{r}_{1}{x}_{2}=-{r}_{1}$$

21b $$\frac{d{x}_{1}}{d{x}_{2}}=\infty \to {r}_{2}{x}_{1}+{r^{\prime} }_{2}{x}_{2}=-{r}_{2}$$

Both of these isoclines are straight lines with positive *x*_1_ and *x*_2_ intercepts depending case of (i) ${r}_{1} > {r^{\prime} }_{1}$ and ${r}_{2} < {r^{\prime} }_{2}$, (ii) ${r}_{1} < {r^{\prime} }_{1}$ and ${r}_{2} > {r^{\prime} }_{2}$, (iii) ${r}_{1} < {r^{\prime} }_{1}$ and ${r}_{2} < {r^{\prime} }_{2}$, (iv) ${r}_{1} > {r^{\prime} }_{1}$ and ${r}_{2} > {r^{\prime} }_{2}$. From time-dependent differential equations, one can see that there are two of three equilibrium population of species depending on the EoS parameters in the competing-species model in Eq. (19). In the first and second cases, there is only two equilibrium point which corresponds to the extinction of at least one of the species in the universe. However, in the cases of third and fourth, there are three equilibrium points in which both species in the universe coexist. This equilibrium population is given by the intersection of the two straight lines ${r^{\prime} }_{1}{x}_{1}^{E}-{r}_{1}{x}_{2}^{E}=-{r}_{1}$ and ${r}_{2}{x}_{1}^{E}+{r^{\prime} }_{2}{x}_{2}^{E}=-{r}_{2}$. Thus these points are given

22 $${x}_{1}^{E}=-\frac{({r}_{1}{r}_{2}+{r^{\prime} }_{1}{r^{\prime} }_{2})}{({r}_{1}{r}_{2}+{r}_{1}{r^{\prime} }_{2})},\quad {x}_{2}^{E}=-\frac{({r}_{1}{r}_{2}+{r^{\prime} }_{1}{r}_{2})}{({r}_{1}{r}_{2}+{r^{\prime} }_{1}{r^{\prime} }_{2})}$$

Stability of the coexistent equilibrium population can be discussed for different cases. We can also obtain trajectories of coupling eq. (19). However, The trajectory will shift without changing its character.

### Generalized equation for nonlinear interactions

We present the generalized equation for coupling interactions as a result. If we assume that there can be non-linear interactions among multiple components in the universe, then we can write a more general interaction equation. To achieve a more general equation, we start to write the interactions that a single component, such as dark energy, can perform first with itself and with other components, respectively.

23a $$\frac{d{x}_{1}}{dt}=r{x}_{1}(1-r^{\prime} {x}_{1})$$

23b $$\frac{d{x}_{1}}{dt}=r{x}_{1}(1-(r^{\prime} {x}_{1}+r^{\prime\prime} {x}_{2}))$$

23c $$\frac{d{x}_{1}}{dt}=r{x}_{1}(1-(r^{\prime} {x}_{1}+r^{\prime\prime} {x}_{2}+r^{\prime\prime\prime} {x}_{3}))$$

and so on…, where *r* and *r* primes are interaction parameters between components. Finally, we can generalize to

24 $$\frac{d{x}_{i}}{dt}={r}_{i}{x}_{i}(1-\sum _{j=1}^{N}{\eta }_{ij}{x}_{j})\quad i=1,\mathrm{...},N$$

where *x*_*i*_ denotes the density of the *i*-th species, i.e., *x*_1_ is the dark energy, *x*_2_ is the dark matter *x*_3_ is the matter and *x*_4_ is the radiation. On the other hand, *r*_*i*_ is its intrinsic growth (or decay) rate and the matrix *η*_*ij*_ is called the interaction matrix. For *i* = 1, *x*_1_ corresponds to dark energy and rate parameter is ${r}_{1}=-3H(1+{w}_{de})$; for *i* = 2, *x*_2_ corresponds to dark matter and its parameter is *r*_2_ = −3*H*(1 + *w*_*dm*_) and so on. On the other hand, matrix elements *η*_*ij*_ are given by ${\eta }_{11}=-\frac{3H{{w}^{^{\prime} }}_{de}(1+{w}_{dm})}{(1+{w}_{dm})}$, ${\eta }_{12}=1$, ${\eta }_{13}=\frac{(1+{w}_{dm})}{(1+{w}_{de})}$, and so on. This equation can be called *generalized interacting equation* for all component such as dark energy, dark matter, radiation etc., and this equation likes the equation of competitive species in biological systems (See refs^60,61^). In other words, this equation for *N* components is the counterpart of the Lotka-Volterra equation in cosmology.

### Interaction dynamics of the dark energy - dark matter - matter

Here, we present the results of the coupling interactions among dark energy, dark matter, and matter. Firstly, to obtain numerical for *N* = 3 we can write the Eq. (24) in the form^61^

25 $$\frac{d{x}_{i}}{dt}={x}_{i}\sum _{j=1}^{3}{\alpha }_{ij}(1-{x}_{j})$$

where *α*_*ij*_ = *r*_*i*_ *η*_*ij*_. Here we consider that EoS for dark energy *w*_*de*_ is a little bit greater than −1 which means the dark energy density will slowly decrease as the universe expand. In order to numerically solve the Eq. (25) we chose 0 > *w*_*de*_ > −1, *w*_*dm*_ ≥ 0 and *w*_*m*_ ≥ 0 that provides the conditions *r*_1_ < 0, *r*_2_ > 0 and *r*_3_ > 0. The parameters *α*_*ij*_ to solve Eq. (25) are given by

26 $${\alpha }_{ij}=(\begin{array}{ccc}-0.5 & -0.1 & 0.1\\ 0.5 & 0.5 & 0.1\\ \mu  & 0.1 & 0.1\end{array})$$

**Figure 2:**
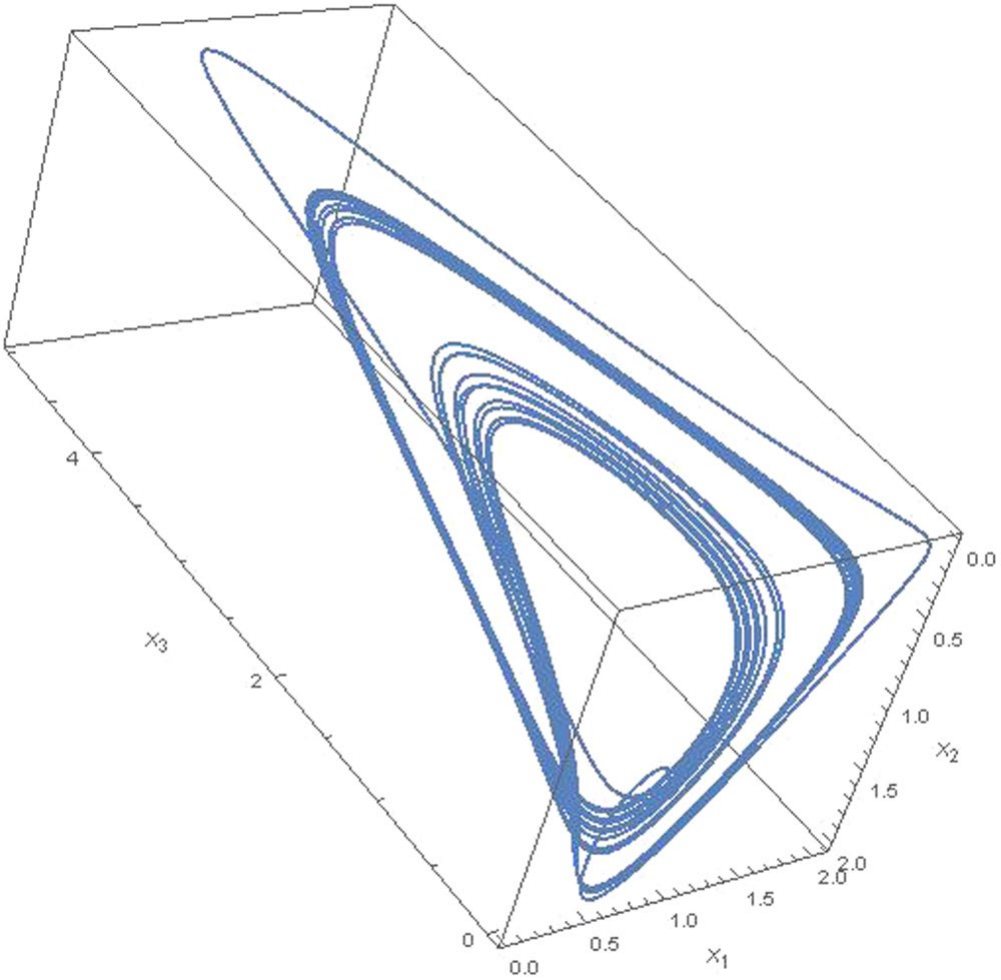
Chaotic attractor for interaction between the dark energy *x*_1_, dark matter *x*_2_ and matter *x*_3_ at *μ* = 1.39 for *w*_*de*_ > −1, *w*_*dm*_ ≥ 0 and *w*_*m*_ ≥ 0 for 5000 time step.

**Figure 3:**
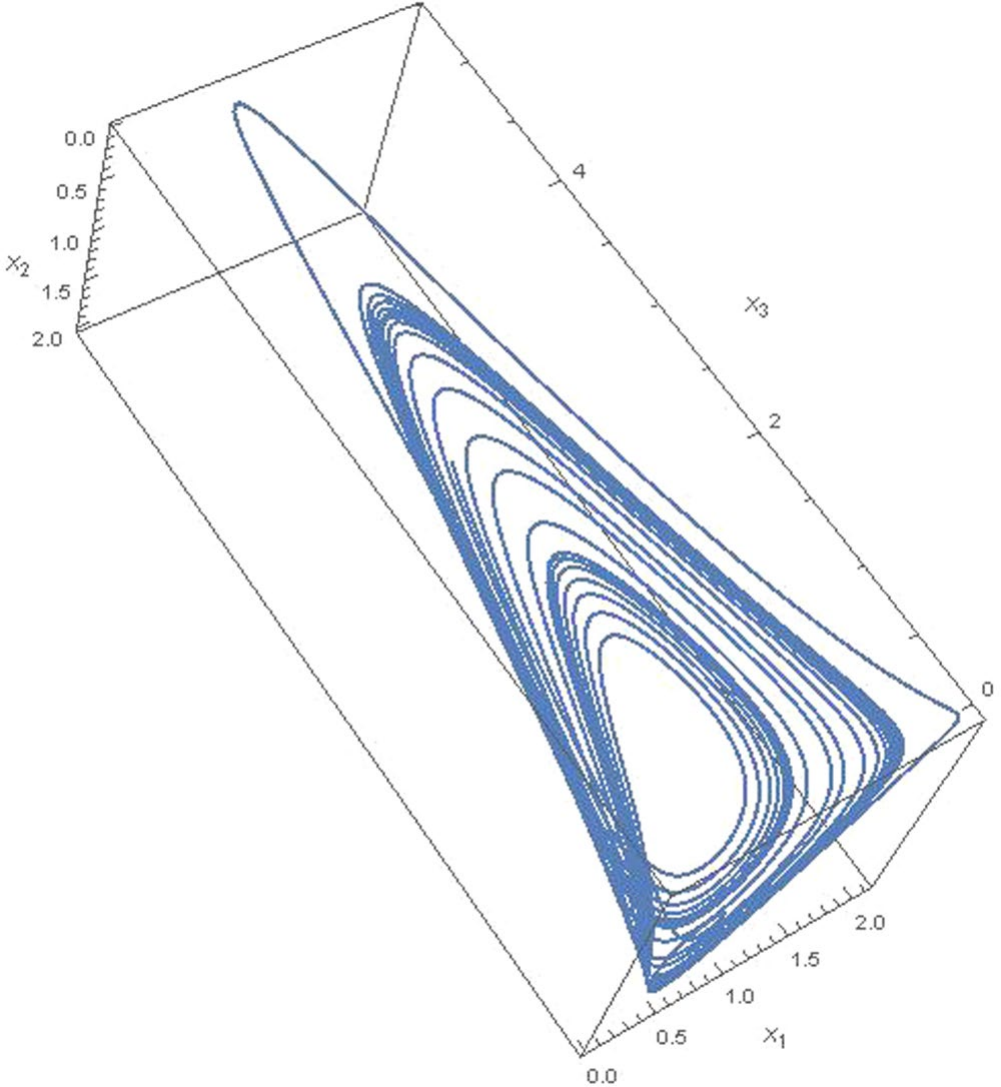
Chaotic attractor for interaction between the dark energy *x*_1_, dark matter *x*_2_ and matter *x*_3_ at *μ* = 1.43 for *w*_*de*_ > −1, *w*_*dm*_ ≥ 0 and *w*_*m*_ ≥ 0 for 5000 time step.

For these *α*_*ij*_ parameters satisfy *r*_1_ = −0.5, *r*_2_ = 1.1 and *r*_3_ = *μ* + 0.2. By using data set (26) we obtain phase space solutions for two different arbitrary *μ* > 0 values. Obtained numerical results for *μ* = 1.39 and *μ* = 1.43 are given in Figs 2 and 3, respectively. In Fig. 2 we set initial values for *μ* = 1.39 at *t* = 0 we set *x*_1_ = 0.2, *x*_2_ = 0.3 and *x*_3_ = 0.14. As it can be clearly seen from Fig. 2 for these initial conditions and data set given in (26) Eq. (25) has a strange attractor. By using TISEAN package program we compute the largest Lyapunov exponent as *λ* = 0.045. On the other hand in Fig. 3 for *μ* = 1.43 at *t* = 0 we set *x*_1_ = 0.3, *x*_2_ = 0.7 and *x*_3_ = 0.1. Figure 3 also shows that dynamics of Eq. (25) has a chaotic attractor with Lyapunov exponent *λ* = 0.091. These strange attractors provide that the dynamics of coupling interactions between dark energy, dark matter and matter is chaotic. These solutions are repeated for different initial and different data set provides the conditions 0 > *w*_*de*_ > −1, *w*_*dm*_ ≥ 0 and *w*_*m*_ ≥ 0 and find similar chaotic behavior in the coupling dynamics. We also note that the numerical solution is independent from the Hubble parameter for mutual interactions between components since it lives in the factor of *α*_*ij*_ = *r*_*i*_ *η*_*ij*_ and is canceled. Thus the parameter *H* does not appears in mutual interactions as well Eq. (8).

Here obtained results shows that chaotic behavior appears in coupling interaction dynamics when dark energy parameter takes 0 > *w*_*de*_ > −1 values. In cosmology, many experimental observations provide that dark energy EoS parameter *w*_*de*_ bigger than minus one for the different era of the universe. Therefore, these observations provide 0 > *w*_*de*_ > −1 supports the idea that the universe may have chaotic behavior in any period. It is well known that if a physical system has a chaotic dynamics it creates an order according to the chaotic dynamics and does not easily leave itself in order. The importance of chaotic dynamics for the universe discuss in next section.

We show that, unlike binary interactions, triple coupling interactions lead to chaotic dynamics. This is, of course, is a very simple model and is based entirely on the idea that the constituents of the universe are transformed to each other through interactions. This simple model, inspired by the competition of species in biological systems, offers remarkable results on the universe dynamics. If we go back to the biological systems, we know that the competing systems for *N* = 3 admit limit cycles behavior. Vano *et al*.^60^ studied the occurrence of chaos in basic Lotka-Volterra models of Lotka-Volterra models of four competing species. Apparently, for *N* ≤ 3 chaos is not possible. However for *N* = 3 it is shown that chaotic behavior can appear in Eq. (25) for unphysical parameters^61^. Contrary to *N* = 3 Lotka-Volterra equations our equation in Eq. (25) shows that the chaotic behavior appears in the universe due to interactions between dark energy, dark matter and matter for realistic parameters.

### Interaction dynamics of the dark energy - dark matter - matter - radiation

Finally we present the results of the coupling interaction between dark energy, dark matter, matter and radiation densities. For these interactions we set 0 > *w*_*de*_ > −1, *w*_*dm*_ ≥ 0, *w*_*m*_ ≥ 0 and *w*_*r*_ ≥ 0. To obtain numerical for *N* = 3 we can write the Eq. (24) in the form^61^

27 $$\frac{d{x}_{i}}{dt}={x}_{i}\sum _{j=1}^{4}{\alpha }_{ij}(1-{x}_{j})$$

where *α*_*ij*_ = *r*_*i*_ *η*_*ij*_. The parameters *α*_*ij*_ to solve Eq. (27) are given by

28 $${\alpha }_{ij}=(\begin{array}{cccc}-0.5 & -0.1 & 0.1 & 0.1\\ -0.5 & 0.5 & 0.1 & 0.2\\ 0.3 & 0.1 & 0.1 & -0.3\\ 0.1 & 0.1 & 0.1 & 0.1\end{array})$$

**Figure 4:**
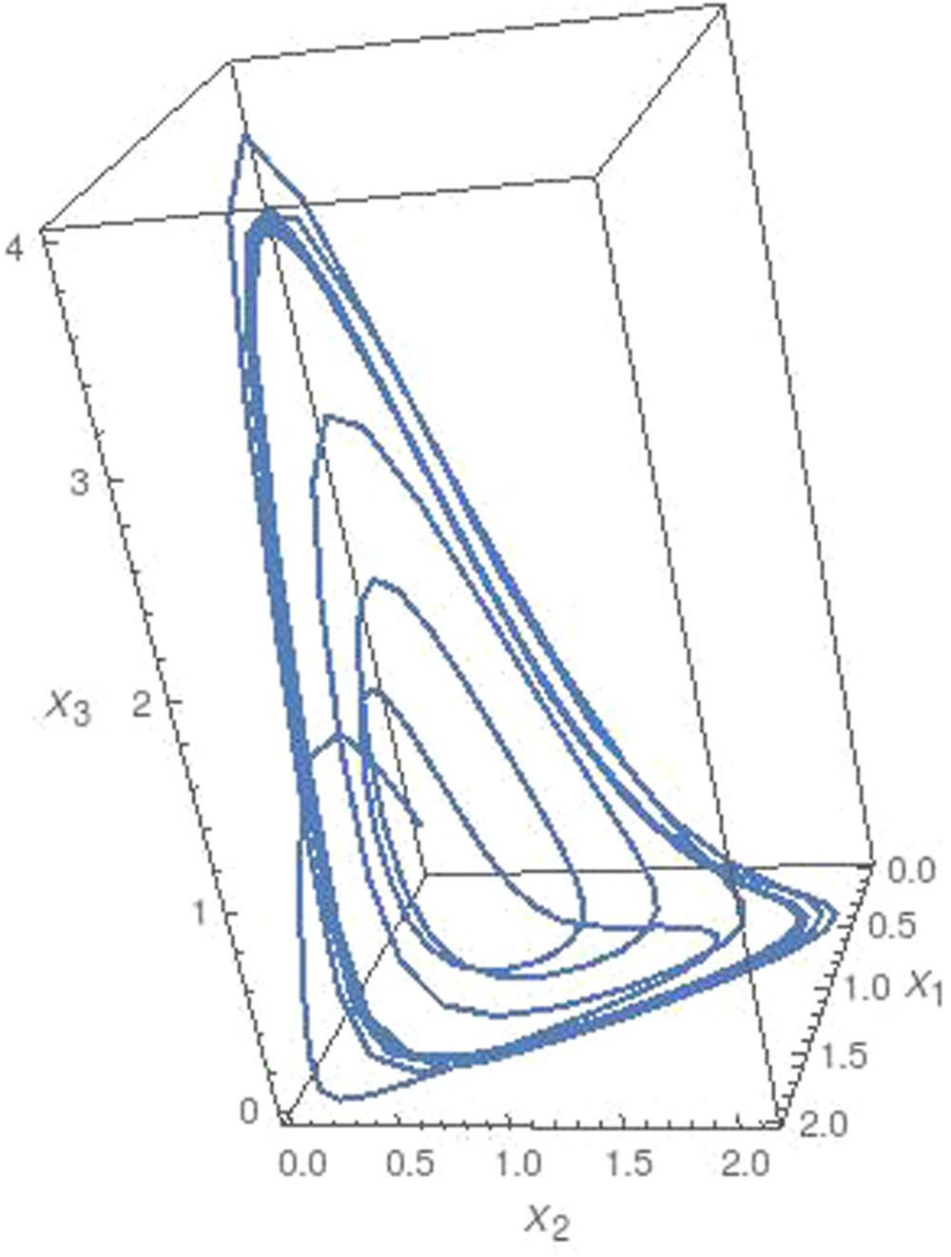
Chaotic attractor for interaction between the dark energy *x*_1_, dark matter *x*_2_, matter *x*_3_ and radiation *x*_4_ for 2000 time steps.

For these *α*_*ij*_ parameters satisfy *r*_1_ = −0.6, *r*_2_ = 0.6, *r*_3_ = 0.2 and *x*_4_ = 0.4. By using initial conditions *x*_1_(0) = 0.21, *x*_2_(0) = 0.35, *x*_3_(0) = 0.11 and *x*_4_(0) = 0.51 at *t* = 0, Eq. (27) for four species is solved. Obtained results is shown in Fig. 4. We also find positive Lyapunov exponent *λ* = 0.011 for these data. It can be clearly shown that coupling interactions for arbitrary but 0 > *w*_*de*_ > −1, *w*_*dm*_ ≥ 0, *w*_*m*_ ≥ 0 values and *w*_*r*_ ≥ 0 and arbitrary *α*_*ij*_ values has a chaotic solutions. Here these solutions are also repeated for different initial and different data set provides the conditions 0 > *w*_*de*_ > −1, *w*_*dm*_ ≥ 0 and *w*_*m*_ ≥ 0 and find similar chaotic behavior in the coupling dynamics.

## Discussion

In this study, we consider nonlinear interactions between components of the universe such as dark energy, dark matter, matter and radiation, and propose a new model to explain the dynamics of the universe. We numerically solve the model by using arbitrary EoS parameters and analyses the time-dependent behavior of densities of these components. Obtained results show that in the presence of the mutual interactions between components, our universe may have chaotic dynamics.

In summary several steps are followed in the study. *In the first step*, we consider only the interaction between dark energy and dark matter, and we show that this interaction can be represented by an equation of Lotka-Volterra type given in Eq. (8) for the conditions *w*_*de*_ < −1, *w*_*dm*_ ≥ 0 and *w*_*m*_ ≥ 0. Results in Fig. 1 show that there is a limit cycle behavior between dark energy and dark matter for *w*_*de*_ < −1, *w*_*dm*_ ≥ 0 and *w*_*m*_ ≥ 0. *In the second step*, we consider the quadratic contribution to the coupling interactions for the conditions *w*_*de*_ < −1, *w*_*dm*_ ≥ 0 and *w*_*m*_ ≥ 0 and we show that these interactions can be given by Eq. (19). I*n the third step*, we consider *N* coupling interactions between components of the universe. We generalized these interaction equations the help of quadratic interactions for arbitrary interaction parameters. We show that the generalized equation can be given in the form of (24) which is similar to the generalized Lotka-Volterra equation^60,61^. *In the fourth step* we consider triple coupling interactions between dark energy, dark matter and matter and we solve numerically Eq. (25) for the data set in (26) for two different arbitrary *μ* > 0 values. Obtained numerical results are shown in Figs 2 and 3 that chaotic behavior appears in the dynamics of the coupling interactions for the conditions 0 > *w*_*de*_ > −1, *w*_*dm*_ > 0, *w*_*m*_ > 0 and and arbitrary *α*_*ij*_ parameter values. *In last step*, we study coupling interactions between different four species such as dark energy, dark matter, matter and radiation. We numerically analyses Eq. (27) for four species by using data in (28). We find in Fig. 4 that for *N* = 4 interactions also has chaotic behavior for the conditions 0 > *w*_*de*_ > −1, *w*_*dm*_ > 0, *w*_*m*_ > 0, *w*_*r*_ > 0 and arbitrary *α*_*ij*_ parameters. In the last two step, EoS parameter for the dark energy is set as *w*_*de*_ > −1 which imply that dark energy density will slowly decreases while the universe expanding.

Here, we focus on the dynamic of the coupling eqs (25) and (27). Therefore, to obtain the numerical solution of these equations, we set arbitrary EoS parameters for components in the interaction matrices (26) and (28). In figures, the mutual changing of the densities are given. When the eqs (25) and (27) are examined, one can see that the interaction dynamics in Figs 2–4 are completely dependent on the EoS parameters and the nonlinear coupling equations which produce chaotic dynamics. Similar results can be found for different initial values and different data set provided the conditions 0 > *w*_*de*_ > −1, *w*_*dm*_ ≥ 0 and *w*_*m*_ ≥ 0. In figures, the global oscillations between densities indicate that different dominant periods exist. Therefore, it may not possible to compare all results in figures with the experimental data available today. However, obtained numerical result in the present study are consistent with the constraints of the SNIa^14–17^, CMB anisotropy^18,19^ and LSS^20–22^ experimental data although we set arbitrary EoS parameters. In order to see the experimental constraints on the our results, for example, it can be looked at the *x*_1_ − *x*_2_ plane in Figs 2 and 3. As it can be seen from these figures that the dark energy density *x*_1_ and dark matter density *x*_2_ change roughly in the intervals $({x}_{1},{x}_{2})\sim \{0.2-1.5\}$ and $({x^{\prime} }_{1},{x^{\prime} }_{2})\sim \{1.5-0.2\}$. This interval compatible with the SNIa data^62^ (Please see Figs 11–15 in this reference). On the other hand, it is possible to find more confidential data interval in the *x*_1_ − *x*_2_ plane when the matter density *x*_3_ is roughly close to zero. It can be seen from figures that there is an intersection between the large *x*_1_ ~ 0.5 − 0.7 and small *x*_2_ ~ 0.3 − 0.5. The presence of this intersection indicates that density dynamics of the coupling equations can be fitted to the experimental data CMB anisotropy^18,19^, LSS^20–22^ and the combination of several data^62^.

This interaction model, inspired by the Lotka-Volterra equation representing the competition of biological species, that the universe has chaotic dynamics. Although this competing model is very simple, it leads to very interesting results. The chaotic universe model not only offers a new perspective and a different scenario from well known popular model for the universe dynamics and its evolution but also may have the potential of solving many important problems of cosmology such as the singularity, cosmic coincidence, big crunch, big rip, horizon, oscillation, emergence of the galaxies, matter distribution and large-scale organization of the universe. We briefly summarize the possible main results of the chaotic universe dynamics: (1) Chaotic universe model has a different scenario from the well-known popular model such as the big-bang and oscillating universe models. According to the chaotic universe model, the universe oscillates in time with chaotic dynamics without repeating itself. In this universe model, there is no singularity, big crunch or big rip. The universe evolves depending on the competing between components. In this scenario, for example; when the dark energy density increases, the universe begins to expand up to a critical dark energy density. However, when the dark energy begins to turn into dark matter or matter, the gravitational force becomes dominant and the universe shrinks again due to gravitational force. This chaotic cycle continues without repeating itself. (2) This scenario solves the cosmic coincidence problem. (3) The model can explain the horizon problem. According to the relativistic physical theories, no information can travel faster than the speed of light. This assumption leads to causality problem. Disconnected regions of the universe cannot have shared any sort of information since they are not in causal contact. It is generally expected that in the absence of common initial conditions that their physical properties would be different. On the other hand, the CMB should not be isotropic if the universe started with even slightly different temperatures in different places. However disconnected regions of the universe may have similar structures and matter distributions even though they have the different initial condition. Moreover, the CMB has the same temperature in the entire sky. The chaotic universe model allows the local interactions between components. These interactions can lead to the similar formations and homogeneity of the CMB in disconnected regions of the universe. (4) Strong local interactions also provide a mechanism for galaxy formations in independent regions. (5) The chaotic universe model allows us to explain why the fractal forms in the universe have emerged from the microcosmos to the macro cosmos at all scales. (6) The model allows us to write equations that can represent the simplest possible dynamics of the universe. (7) The model suggests that the universe evolved by scaling itself in a similar way. (8) The model connects between dynamics of the competing species in biological systems and dynamics of the time evolution of the universe. (9) Finally, the model presents a new perspective and a different scenario for the universe dynamics and evolution, unlike well known popular models.

## Methods

In this study, in order to derive interaction equations, we consider Friedman-Robertson-Walker space-time. In this space-time, the line elements are given by

29 $$d{s}^{2}=-d{t}^{2}+a{(t)}^{2}\mathop{\sum _{i=1}}\limits^{3}{(d{x}^{i})}^{2}$$

where *a*(*t*) is the scale factor of the three-dimensional flat space, *i* indicates the spatial components. FRW equations due to the metric (29) are given by

30 $${H}^{2}=\frac{{\kappa }^{2}}{3}\rho ,\quad \dot{H}=-\frac{{\kappa }^{2}}{2}(\rho +p)$$

where *κ*^2^ = 8*πG* is the gravitational constant, $H\equiv \frac{\dot{a}}{a}$ is Hubble rate, *ρ* is energy density and *p* is pressure. The energy density *ρ* and pressure *p* satisfy continuity equation, i.e, energy conservation equation

31 $$\dot{\rho }+3H(\rho +p)=0$$

where over-dot indicates the time derivative. The relation between *ρ* and *p* is given by

32 $$p=w\rho $$

where *w* is EoS parameter which is constant and equal to exactly −1 for the FRW framework. For the single fluid the energy conservation (31) is given in known form

33 $$\dot{\rho }+3H\rho (1+w)=0.$$

In this study we derived the coupling equations in (8) based on Eq. (33). By adding quadratic interaction into the Eq. (5) self-interacting equations in Eq. (19) are derived. On the other hand, with help of the eqs (5) and (8) generalized interaction equation in (24) is obtained for *N* components. The special cases of the Eq. (24) are given in (25) and (27). Finally, the numerical solutions of the eqs (8), (25) and (27) are computed by using the Mathematica package program and all figures are plotted with help of the same package. The largest Lyapunov exponents are computed with help of the TISEAN package program.
